# Post-Diagnostic Aspirin Use in Breast Cancer Treatment: A Systematic Review and Meta-Analysis of Survival Outcomes with Trial Sequential Analysis Validation

**DOI:** 10.3390/diagnostics15010044

**Published:** 2024-12-27

**Authors:** Po-Huang Chen, Tung-Lung Yang, Hong-Jie Jhou, Hsu-Lin Lee, Ming-Shen Dai

**Affiliations:** 1 Division of Hematology and Oncology, Department of Internal Medicine, Tri-Service General Hospital, National Defense Medical Center, Taipei 114, Taiwan; chenpohuang@hotmail.com (P.-H.C.); doc10897@mail.ndmctsgh.edu.tw (T.-L.Y.);; 2 Department of Neurology, Changhua Christian Hospital, Changhua 500, Taiwan; xsai4295@gmail.com

**Keywords:** aspirin, breast cancer, meta-analysis

## Abstract

**Background**: Breast cancer is a leading cause of cancer-related mortality in women. Aspirin, an affordable anti-inflammatory drug, may have anticancer effects, but its impact on survival outcomes after breast cancer diagnosis remains unclear. This meta-analysis evaluates the role of post-diagnostic aspirin use in breast cancer management. **Methods**: A systematic review and meta-analysis were conducted using PubMed, EMBASE, and Cochrane Library databases. Twenty studies involving 141,251 participants were included. Survival outcomes assessed were disease-free survival (DFS), overall survival (OS), and breast cancer-specific mortality. Trial sequential analysis (TSA) was used to evaluate the sufficiency of cumulative evidence. **Results**: Post-diagnostic aspirin use was not significantly associated with DFS (HR: 0.88; 95% CI: 0.69–1.11) or OS (HR: 0.89; 95% CI: 0.74–1.07). However, a significant reduction in breast cancer-specific mortality was observed (HR: 0.77; 95% CI: 0.63–0.93). TSA confirmed that the evidence supporting this association is sufficient. **Conclusions**: Post-diagnostic aspirin use significantly reduces breast cancer-specific mortality, but it does not improve DFS or OS. These findings underscore the potential therapeutic role of aspirin in breast cancer management. Further randomized controlled trials are needed to validate these results and determine optimal dosing regimens for post-diagnostic use.

## 1. Introduction

Breast cancer is the most commonly diagnosed cancer and a leading cause of cancer-related mortality among women worldwide. In 2020, approximately 2.26 million new cases of breast cancer were diagnosed globally, resulting in an estimated 685,000 deaths [1]. Given this significant impact on global morbidity and mortality, identifying effective treatments to improve survival outcomes in patients with breast cancer is a high priority in oncology research.

Aspirin, a widely accessible and cost-effective non-steroidal anti-inflammatory drug (NSAID), has garnered attention for its potential anticancer properties. While the United States Preventive Services Task Force (USPSTF) has recommended aspirin for the primary prevention of colorectal cancer, research has expanded to investigate its applicability in other cancers, including breast cancer [2]. The mechanisms by which aspirin may exert anticancer effects are dose-dependent [3]. At low doses (75–100 mg daily), typically used for cardiovascular prevention, aspirin’s primary mechanism is through irreversible platelet inhibition, which may limit cancer metastasis by reducing platelet-mediated protection of circulating tumor cells [4]. At higher anti-inflammatory doses (>325 mg daily), aspirin additionally inhibits cyclooxygenase-2 (COX-2), an enzyme commonly overexpressed in aggressive breast cancers and associated with poorer prognosis [5]. This dual mechanism of action distinguishes aspirin from other NSAIDs, which typically require higher doses for both antiplatelet and anti-inflammatory effects. Beyond these pathways, aspirin also inhibits signaling pathways such as PI3K/Akt/mTOR, which are critical in cellular proliferation and survival, further supporting its potential to limit tumor growth and spread [6].

Observational studies have provided promising evidence for a reduction in mortality among breast cancer survivors who use aspirin regularly. Data from trials, although not primarily focused on cancer outcomes, have also shown incidental reductions in cancer incidence and metastasis among aspirin users, especially for adenocarcinomas, which include breast cancer [7]. For instance, pooled analyses from large trials indicate that aspirin use is associated with a reduced risk of distant metastasis in patients with cancer [8]. Meta-analyses of observational studies specifically focusing on breast cancer have similarly suggested that post-diagnostic aspirin use may be associated with reduced breast cancer-specific mortality [9].

However, results from previous meta-analyses are inconsistent, and not all studies have shown a clear benefit of post-diagnostic aspirin use. Some earlier meta-analyses included both pre- and post-diagnostic aspirin use, potentially making it challenging to isolate the specific effects of aspirin when taken after a breast cancer diagnosis [10]. Moreover, several analyses are outdated, with the literature searches extending only to 2014, limiting their relevance given the increase in research on this topic over the past decade. Inconsistent findings from recent studies further underscore the need for a rigorous, updated synthesis of evidence to clarify the role of post-diagnostic aspirin use in breast cancer management.

In light of these gaps, this study aims to conduct a comprehensive meta-analysis focused exclusively on post-diagnostic aspirin use in patients with breast cancer. Given the inconsistencies in previous meta-analyses, we have also incorporated trial sequential analysis (TSA) to assess the sufficiency of the cumulative evidence and to determine whether the available data are conclusive or if additional studies are required to confirm the effects of post-diagnostic aspirin use on breast cancer outcomes. This study is particularly important as it provides a focused evaluation of aspirin’s therapeutic role post-diagnosis, offering updated evidence that may inform clinical decision-making and future research on optimizing breast cancer management strategies.

## 2. Materials and Methods

### 2.1. Study Design and Data Sources

This meta-analysis and systematic review was conducted in accordance with PRISMA guidelines [11] (Table S1). A comprehensive literature search was performed in PubMed, EMBASE, and the Cochrane Library to identify studies on post-diagnostic aspirin use in patients with breast cancer. The search strategy, detailed in Table S2, included the keywords “aspirin” and “breast cancer”, with no restrictions on publication date or language. Our research will be preregistered on the Open Science Framework at https://osf.io/pyh8d (accessed on 1 December 2024).

### 2.2. Eligibility Criteria

Studies were included if they met the following criteria: (1) involved patients diagnosed with invasive breast cancer; (2) investigated post-diagnostic aspirin use; (3) reported on survival outcomes, including disease-free survival (DFS), overall survival (OS), and breast cancer-specific mortality; and (4) provided sufficient data to calculate hazard ratios. Exclusion criteria included studies that focused on pre-diagnostic aspirin use, lacked survival data, or reported only on breast cancer incidence. Reasons for excluding studies are listed in Table S3.

### 2.3. Study Selection Process

The initial search results were imported into reference management software (Zotero 6.0, Fairfax, VA, USA), and duplicates were removed. Titles and abstracts were screened independently by two reviewers to assess relevance. Full texts of potentially eligible studies were then reviewed in detail to confirm eligibility. Any discrepancies were resolved through discussion with a third reviewer.

### 2.4. Data Extraction

Data extraction was conducted independently by two reviewers, P.H.C. and T.L.Y., using a structured form in Google Sheets to systematically capture essential information from each study. Key data points included study characteristics, patient demographics, cancer-specific data, and survival outcomes (DFS, OS, and breast cancer-specific mortality). Study characteristics encompassed publication details, study location, design, sample size, recruitment period, follow-up duration, and aspirin dosage. Patient demographics included age, sex, race, smoking status, menopausal status, and clinical attributes. Cancer-specific information covered staging, grading, hormone receptor (HR+) and HER2 status, lymph node involvement, and treatments received, including radiotherapy, chemotherapy, hormone therapy, and surgery. Any discrepancies during data extraction were resolved through discussion with a third reviewer, M.S.D., to ensure accuracy and consistency.

### 2.5. Quality Assessment

The quality of included studies was assessed according to study design by two independent reviewers (P.H.C. and T.L.Y.), with discrepancies resolved through discussion with a third reviewer (M.S.D.). For randomized controlled trials (RCTs), we used the Cochrane Risk of Bias 2.0 (ROB 2.0) tool [12], which evaluates bias across domains including the randomization process, deviations from intended interventions, missing outcome data, measurement of outcomes, and selective reporting. For non-randomized studies, we employed the Risk Of Bias In Non-randomized Studies of Interventions (ROBINS-I) tool [13], which assesses potential bias in pre-intervention, at-intervention, and post-intervention stages. For studies reporting both NSAID and aspirin data, only aspirin-specific results were extracted to maintain analytical clarity.

### 2.6. Meta-Regression Analysis

Meta-regression analyses were conducted to explore potential sources of heterogeneity and assess the impact of various study-level covariates on survival outcomes, including DFS, OS, and breast cancer-specific mortality. Covariates analyzed included study publication year, mean age of the study population, and proportions of patients with hormone receptor-positive status, HER2-positive status, postmenopausal status, and Stage I breast cancer. Mixed-effects meta-regression models were applied, with coefficients and corresponding 95% confidence intervals reported for each covariate.

### 2.7. Statistical Analysis

Survival data were analyzed to evaluate the impact of post-diagnostic aspirin use on DFS, OS, and breast cancer-specific mortality. To assess potential publication bias, funnel plots and Egger’s test were used [14]. Statistical heterogeneity was evaluated with the I^2^ statistic and Cochrane’s Q test [15]. Both fixed- and random-effects models were applied to account for inter-study variability, enhancing the robustness of the results. Meta-analyses focused on pairwise comparisons, with hazard ratios (HRs) and 95% confidence intervals (CIs) calculated to compare survival outcomes across studies. All meta-analyses were conducted using R version 4.4.1 with the meta package [16].

In meta-analyses, cumulative evidence can increase the risk of type I and type II errors, especially when data are sparse or significance testing is repeated as new studies are added. To mitigate these risks and control the overall false-positive rate, TSA was applied [17]. TSA adjusts the required information size and establishes boundaries to determine whether the evidence is conclusive or if further studies are warranted. This analysis was conducted in Stata using the metacumbounds package [18]. The analysis was performed with a significance level (alpha) of 5%, a power of 80%, and a conservatively set relative risk reduction of 5%. The required information size (RIS) was calculated based on the observed data variance, and the default fixed-effects model was used for boundary setting. The heterogeneity adjustment was not included in the RIS calculation.

## 3. Results

### 3.1. Study Selection and Inclusion Summary

**Figure 1:**
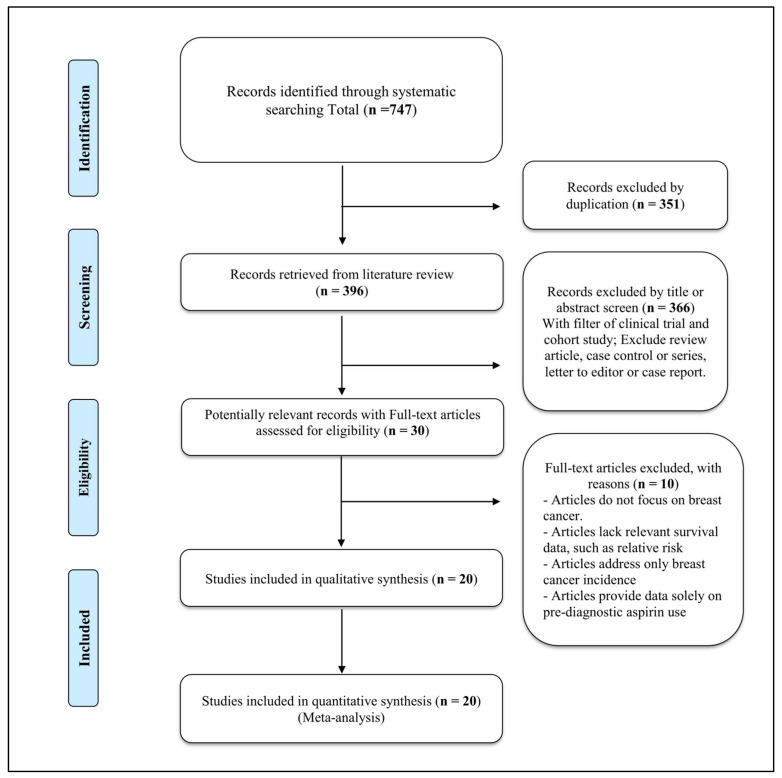
PRISMA flow diagram of study selection process.

The PRISMA flow diagram in Figure 1 outlines the study selection process. A total of 747 records were identified through systematic searching, with initial exclusions for duplicates (*n* = 351) and irrelevant titles/abstracts (*n* = 366). After these exclusions, 30 full-text articles were assessed for eligibility. Of these, 10 were excluded for reasons such as a lack of focus on breast cancer, absence of relevant survival data, focus solely on breast cancer incidence, or pre-diagnostic aspirin data only (Table S3). This process yielded 20 studies for inclusion in both qualitative and quantitative synthesis.

### 3.2. Study and Participant Characteristics

Our systematic review included twenty studies [19,20,21,22,23,24,25,26,27,28,29,30,31,32,33,34,35,36,37,38], comprising 141,251 participants across diverse research settings. This study’s designs were predominantly observational (18 cohort studies) with two RCTs, reflecting the challenges of conducting interventional studies in this population.

Sample sizes varied considerably, from focused clinical cohorts (*n* = 222) to large population-based studies (*n* = 34,188), with a median of 3089 participants. The studies spanned a 34-year period (1986–2020), with 75% conducted after 2000, indicating growing research interest in aspirin’s therapeutic potential. Follow-up durations ranged from 2.6 to 8.3 years (median 6.1 years), providing adequate observation periods for assessing long-term outcomes. Analysis of aspirin dosing strategies revealed that 85% of studies employed low-dose regimens (75–81 mg/day), aligning with cardiovascular prevention protocols. However, the heterogeneity in dosing (ranging from 75 to 325 mg/day) underscores the uncertainty regarding optimal dosing for cancer-specific outcomes (Table 1).

Demographic analysis (Table 2) showed a predominantly female population with substantial age diversity, ranging from younger cohorts (30–55 years) to older postmenopausal groups (>65 years). The median age across studies was 62.4 years, with 71.8% of participants being postmenopausal where reported. While racial distribution was not consistently reported, studies documenting ethnicity showed a predominance of Caucasian participants (ranging from 28.9% to 94.2%), highlighting potential limitations in population diversity. Among studies reporting body mass index (BMI), 32.8% of participants had a BMI > 30, and smoking status, when documented, showed non-smokers comprising 58.2% of participants.

Cancer-specific characteristics (Table 3) revealed that hormone receptor (HR)-positive disease predominated, with rates ranging from 70.8% to 100% across studies. HER2 status was reported in 40% of studies, with positivity rates varying from 9% to 34.8%. Stage distribution showed a predominance of early-stage disease, with 42.5% Stage I, 44.7% Stage II, and 12.8% Stage III across studies reporting staging data. Treatment patterns demonstrated comprehensive management: radiotherapy (57.2%), chemotherapy (48.2%), hormone therapy (66.3%), and surgery (91.1%) among studies reporting these interventions.

### 3.3. Forest Plot Analysis and TSA of Survival Outcomes

**Figure 2:**
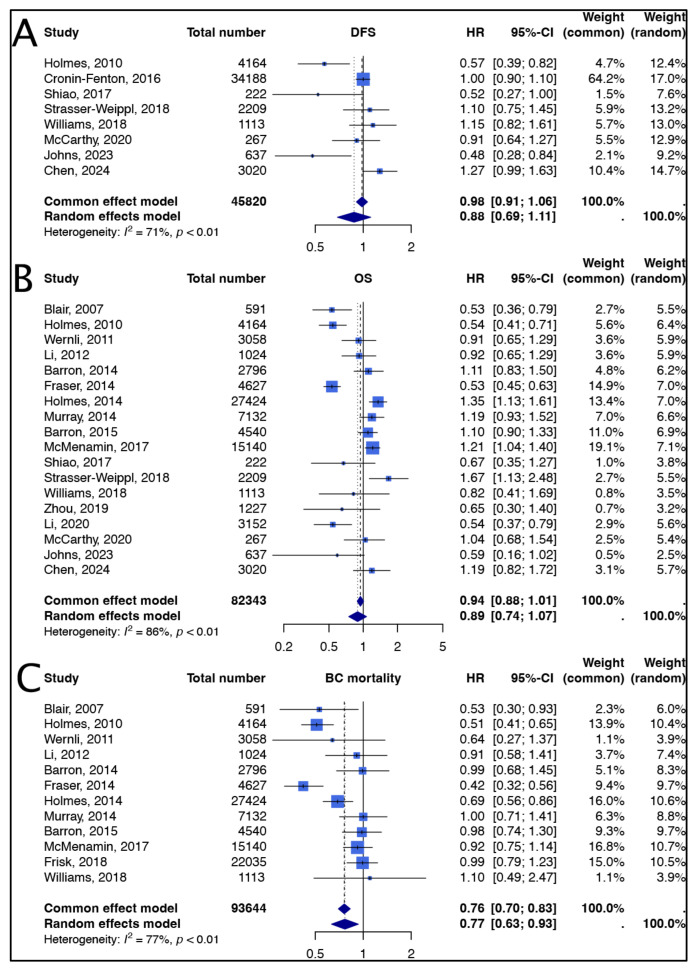
Forest plot analysis of disease-free survival (**A**), overall survival (**B**), and breast cancer-specific mortality (**C**) [19,20,21,22,23,24,25,26,27,28,29,30,31,32,33,34,35,36,37,38].

**Figure 3:**
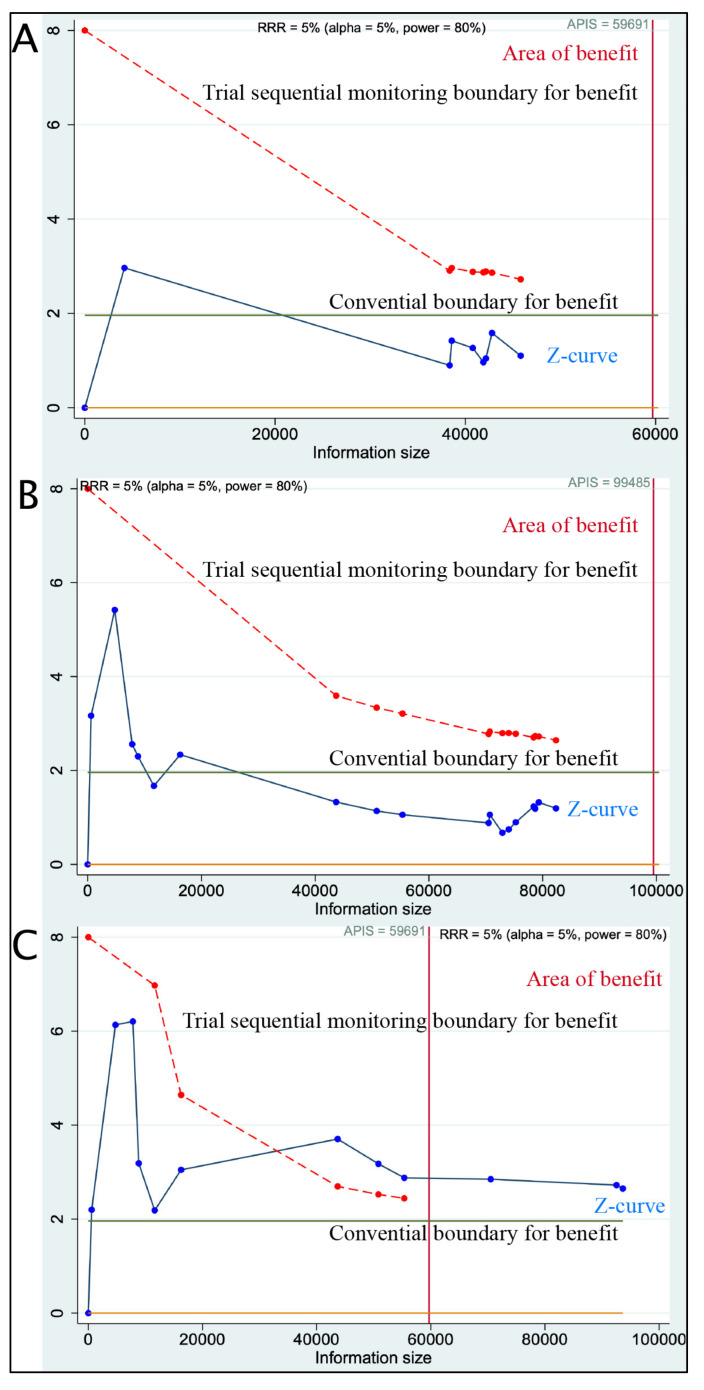
Trial sequential analysis of disease-free survival (**A**), overall survival (**B**), and breast cancer-specific mortality (**C**).

The impact of post-diagnostic aspirin use on DFS, OS, and breast cancer-specific mortality is presented in Figure 2, with TSA in Figure 3 evaluating the sufficiency of cumulative evidence for each outcome.

For DFS, based on eight studies comprising 45,820 patients, the pooled HR under the common effect model was 0.98 (95% CI: 0.91–1.06), while the random-effects model yielded an HR of 0.88 (95% CI: 0.69–1.11) (Figure 2A). These results suggest no statistically significant association between post-diagnostic aspirin use and DFS. Substantial heterogeneity was observed across studies (I^2^ = 71%, *p* < 0.01), indicating variability in DFS outcomes among studies, possibly due to differences in patient characteristics and study designs. In TSA, the Z-curve did not cross the monitoring boundary for benefit, indicating that current evidence is insufficient to confirm a significant effect of aspirin use on DFS. This suggests a need for additional studies to reach a definitive conclusion (Figure 3A).

For OS, based on eighteen studies with 82,343 patients, the HR for the common effect model was 0.94 (95% CI: 0.88–1.01) (Figure 2B), and for the random-effects model, it was 0.89 (95% CI: 0.74–1.07). While these results hint at a trend toward improved OS with aspirin use, the association did not reach statistical significance. Heterogeneity for OS was high (I^2^ = 86%, *p* < 0.01), reflecting considerable variability across studies. Contributing factors may include differences in dosage, treatment duration, and patient baseline characteristics. In TSA, the Z-curve also failed to cross the sequential monitoring boundary, indicating that the cumulative evidence does not conclusively demonstrate the benefit of aspirin use on OS. Further research with rigorous control of confounding factors is needed to establish conclusive evidence for this outcome (Figure 3B).

In contrast, for breast cancer-specific mortality, based on twelve studies with 93,644 patients, aspirin use was associated with a statistically significant reduction in mortality. The HR was 0.76 (95% CI: 0.70–0.83) (Figure 2C) in the common effect model and 0.77 (95% CI: 0.63–0.93) in the random-effects model. Although substantial heterogeneity was present (I^2^ = 77%, *p* < 0.01), likely due to differences in population characteristics and treatment protocols, the findings consistently indicated a benefit of post-diagnostic aspirin use in reducing breast cancer-specific mortality. In TSA, the Z-curve for breast cancer-specific mortality crossed the trial sequential monitoring boundary for benefit, suggesting that the current cumulative evidence is sufficient to indicate a statistically significant reduction in breast cancer-specific mortality with post-diagnostic aspirin use (Figure 3C).

### 3.4. Risk of Bias and Publication Bias Assessment

The risk of bias for included studies was assessed using ROB 2.0 for randomized controlled trials and ROBINS-I for non-randomized studies, as shown in Figure S1. The ROB 2.0 assessment indicated a generally low risk across domains, with some concerns in areas related to the randomization process and deviations from intended interventions. For non-randomized studies, assessed using ROBINS-I, the majority exhibited a low risk of bias, although moderate to serious risks were noted in domains related to confounding, missing data, and classification of interventions.

Publication bias was evaluated using funnel plots and Egger’s test, as illustrated in Figure S2. The funnel plots for all primary outcomes appeared symmetrical, and Egger’s tests were not statistically significant, indicating no evidence of publication bias across the studies.

### 3.5. Result of Meta-Regression Analysis

The meta-regression analyses revealed no significant associations between any of the examined covariates (including study publication year, the mean age of study population, and proportions of patients with specific characteristics) and survival outcomes. This suggests that the observed heterogeneity in treatment effects may be attributed to complex interactions of multiple factors rather than single identifiable sources. The coefficients and detailed findings are presented in Table 4, providing additional insights into the complexity of treatment responses across different patient populations.

## 4. Discussion

This meta-analysis aimed to evaluate the impact of post-diagnostic aspirin use on survival outcomes in patients with breast cancer, specifically examining DFS, OS, and breast cancer-specific mortality. Based on an analysis of twenty studies involving 141,251 participants, our findings indicate that post-diagnostic aspirin use was not significantly associated with DFS or OS, despite a trend toward improved OS. However, a statistically significant reduction in breast cancer-specific mortality was observed among aspirin users. TSA further reinforced these findings, indicating that the cumulative evidence is sufficient to support the benefit of post-diagnostic aspirin use in reducing breast cancer-specific mortality.

### 4.1. Underlying Biological Mechanisms

Several biological mechanisms likely underpin aspirin’s selective effect on breast cancer-specific mortality. Primarily, aspirin’s COX-2 inhibition and anti-inflammatory properties specifically target cancer-related inflammation pathways. This mechanism affects key metastatic processes, including the promotion of the epithelial-to-mesenchymal transition [39], immune evasion, and angiogenesis [40], potentially explaining its effectiveness in preventing distant metastasis without significantly impacting local recurrence. Additionally, aspirin’s antiplatelet properties play a crucial role in reducing metastatic spread by interfering with platelets’ protection of circulating tumor cells from immune detection [41].

### 4.2. Age-Dependent Effects on Mortality Outcomes

Previous studies have demonstrated that the divergent effects between breast cancer-specific mortality and survival outcomes (DFS and OS) can be attributed to age-related factors [42]. The differential impact of aspirin appears to be modulated by the complex relationship between aging and inflammation. Notably, older individuals and patients with cancer characteristically exhibit chronic low-grade inflammation and compromised immune responses, potentially attenuating the therapeutic efficacy of aspirin’s anti-inflammatory properties. Prior research has also suggested that aspirin may interfere with beneficial inflammatory responses against tumors, specifically in older adults [43]. This age-dependent mechanism provides a plausible explanation for our current findings, where post-diagnostic aspirin use demonstrated a significant reduction in breast cancer-specific mortality without corresponding improvements in overall survival. The selective efficacy observed in our study aligns with these previous mechanistic insights, suggesting that age-related physiological changes may create a differential treatment response pattern. While our meta-regression analysis did not show statistically significant associations between age and survival outcomes, the consistently positive coefficients across all endpoints suggest a trend towards greater benefit from aspirin use in younger patients, though this observation requires further validation in future studies.

### 4.3. Disease Stage and Treatment Efficacy

The disease stage at diagnosis also significantly influences aspirin’s effectiveness. Evidence suggests that aspirin exerts more pronounced effects in patients with early-stage or localized breast cancer, possibly through inhibition of tumor growth pathways [44] and reduction of metastatic potential [45]. This mechanism could explain the observed decrease in breast cancer-specific mortality, as early-stage patients are less likely to develop metastatic disease. However, in patients with advanced cancer or significant comorbidities, aspirin’s beneficial effects on cancer-specific mortality may not translate into improved overall survival, as their prognosis is often determined by factors beyond cancer progression alone. Interestingly, our meta-regression analysis exploring the relationship between Stage I disease proportion and survival outcomes showed coefficients close to zero across all endpoints, suggesting that aspirin’s effects might be consistent across different disease stages, contrary to previous individual studies. This finding highlights the complexity of stage-dependent responses and indicates that the benefits of aspirin may not be limited to specific disease stages.

### 4.4. Hormone Receptor Status and Treatment Response

Hormone receptor status emerges as another critical factor frequently proposed to explain the selective mortality benefit of aspirin. Previous studies have demonstrated stronger associations between NSAID use and reduced breast cancer-specific mortality in ER-positive patients [46]. This effect has been hypothesized to stem from aspirin’s ability to reduce serum estrogen levels, thereby inhibiting estrogen-responsive tumor growth [47]. However, our meta-regression analysis did not reveal significant associations between hormone receptor status and survival outcomes, and the coefficients showed inconsistent trends across variables. These findings suggest that the associations observed in prior studies may not fully explain the observed outcomes in our analysis. Further investigation is needed to clarify the interplay between hormone receptor status and the therapeutic effects of post-diagnostic aspirin use.

### 4.5. Comparative Analysis with Previous Studies

Our findings both align with and differ from previous meta-analyses in several important ways [48]. Consistent with prior meta-analyses, our findings support a significant association between post-diagnostic aspirin use and reduced breast cancer-specific mortality. While Baker et al. included a larger number of initial studies, our analysis applied more stringent inclusion criteria by excluding studies focusing on pre-diagnostic aspirin use, thereby providing a more focused evaluation of aspirin’s therapeutic rather than preventive effects. This methodological decision, although resulting in fewer included studies, offers clearer clinical implications for post-diagnostic treatment decisions. Furthermore, our study is the first to employ TSA in this context, providing a robust framework for evaluating the sufficiency of evidence specifically for post-diagnostic aspirin use.

### 4.6. Study Limitations and Future Directions

Despite these strengths, several limitations should be acknowledged. One major limitation is the substantial heterogeneity among the included studies, driven by differences in study designs, aspirin dosages, follow-up durations, and patient populations. This variability introduces challenges in achieving consistent associations across outcomes such as DFS and OS and may weaken the overall generalizability of the findings. Additionally, our meta-analysis primarily relies on observational studies and healthcare database analyses rather than RCTs, which are less prone to bias. The lack of direct, high-quality RCTs on post-diagnostic aspirin use in breast cancer limits the strength of causal inferences and may increase susceptibility to confounding factors inherent in observational data. Moreover, many studies used self-reported aspirin use, often based on retrospective recall, which could introduce recall bias and limit the accuracy of exposure assessment. Healthcare databases, while valuable for large-scale data, can further contribute to inconsistencies due to variations in patient adherence, undocumented aspirin usage, and imprecise dosing information.

## 5. Conclusions

In conclusion, this meta-analysis demonstrates that post-diagnostic aspirin use significantly reduces breast cancer-specific mortality, although no benefits were observed for disease-free survival or overall survival. While these results are promising, future well-designed randomized controlled trials are essential to determine optimal dosing strategies and identify patient populations most likely to benefit.

## Figures and Tables

**Table 1:** Study Characteristics Overview.

First Author, Year	Study Type	Sample Size	Median Follow-Up	Aspirin Dose	Population Characteristics	Country	Years of Recruitment	Period
Blair, 2007 [19]	Cohort study	591	Median follow-up of 8.3 years	Aspirin	Postmenopausal patients with invasive breast cancer	USA	1986–1992	Post-diagnostic
Holmes, 2010 [20]	Cohort study	4164	NA	Aspirin	Patients with stage I–III breast cancer	USA	1976–2002	Post-diagnostic
Wernli, 2011 [21]	Cohort study	3058	Mean follow-up of 7.2 years	Aspirin	Patients with primary, invasive breast cancer	USA	1988–1999	Post-diagnostic
Li, 2012 [22]	Cohort study	1024	NA	Low-dose aspirin	Patients with primary, invasive breast cancer	USA	1996–2001	Pre- and post-diagnostic
Barron, 2014 [23]	Cohort study	2796	NA	Aspirin (75 mg for 85.7% of prescriptions, 300 mg for 10.8%)	Patients with stage I–III breast cancer	Ireland	2001–2006	Pre- and post-diagnostic
Fraser, 2014 [24]	Cohort study	4627	Median follow-up of 5.7 years	Low-dose aspirin, 75 mg	Patients diagnosed with primary invasive breast cancer	UK	1993–2008	Pre- and post-diagnostic
Holmes, 2014 [25]	Cohort study	27424	Median follow-up of 2.6 years	Low-dose aspirin (75 mg or 160 mg)	Patients with a first-incident breast cancer diagnosis	Sweden	2005–2009	Post-diagnostic
Murray, 2014 [26]	Cohort study	9817	Mean follow-up of 6.9 years	Low-dose aspirin, mainly 75 mg	Patients diagnosed with primary invasive breast cancer	UK	1998–2007	Post-diagnostic
Barron, 2015 [27]	Cohort study	4540	NA	Aspirin (<150 mg/day for 95.5% of cohort, 150 mg for 4.5%)	Patients aged 50–80 with stage I–III breast cancer	Ireland	2001–2011	Post-diagnostic
Cronin-Fenton, 2016 [28]	Cohort study	34188	Median follow-up of 7.1 years	Low-dose aspirin	Patients with stage I–III breast cancer	Denmark	1996–2008	Pre- and post-diagnostic
McMenamin, 2017 [29]	Cohort study	15140	Mean follow-up of 4 years	Low-dose aspirin	Patients with primary breast cancer	UK	2009–2012	Pre- and post-diagnostic
Shiao, 2017 [30]	Cohort study	222	Median follow-up of 41.3 months	Low-dose aspirin	Patients with stage II–III triple-negative breast cancer	USA	1998–2016	Post-diagnostic
Frisk, 2018 [31]	Cohort study	22035	Median follow-up of 3.8 years	Aspirin (75 mg or 160 mg)	Patients with stage I–III breast cancer	Sweden	2006–2012	Pre- and post-diagnostic
Strasser-Weippl, 2018 [32]	Trial	2209	Median follow-up of 4.1 years	Low-dose aspirin, max 81 mg	Postmenopausal patients with hormone receptor-positive early breast cancer	Canada	2003–2008	Post-diagnostic
Williams, 2018 [33]	Cohort study	1113	Mean follow-up of 64.5 months	Aspirin	Patients with primary operable breast cancer (stage I–III)	USA	2005–2013.	Pre- and post-diagnostic
Zhou, 2019 [34]	Cohort study	1227	Median follow-up of 27 months	Aspirin (81 mg or 325 mg)	Patients with breast cancer and PIK3CA mutations	USA	2002–2013	Pre- and post-diagnostic
Li, 2020 [35]	Cohort study	3152	NA	Aspirin	Patients with breast cancer and aged ≥65 years	USA	2016–2016	Pre- and post-diagnostic
McCarthy, 2020 [36]	Cohort study	267	NA	Aspirin (81 mg for 51.9%, 325 mg for 27.8%)	Patients with hormone receptor-positive, HER2-negative (HR+/HER2−) metastatic breast cancer	USA	2009–2016	Post-diagnostic
Johns, 2023 [37]	Cohort study	637	Median follow-up of 3.8 years	Low-dose aspirin	Patients with breast cancer and residual disease after neoadjuvant chemotherapy	USA	2005–2008	Post-diagnostic
Chen, 2024 [38]	Trial	3020	Median follow-up of 33.8 months	Aspirin, 300 mg daily	Patients with high-risk HER2-negative breast cancer post-treatment	USA	2017–2020	Post-diagnostic

**Table 2:** Participant demographics and clinical profiles.

First Author, Year	Age (Mean, yrs)	Sex (Female, %)	Race (Caucasian)	BMI > 30 (%)	Non-Smoker (%)	Postmenopausal (%)
Blair, 2007 [19]	Range (55–69)	100%	NA	NA	NA	100%
Holmes, 2010 [20]	Range (30–55)	100%	NA	NA	NA	68.86%
Wernli, 2011 [21]	Range (18–65)	100%	NA	19.82%	50.80%	NA
Li, 2012 [22]	58.4	100%	91.6	NA	NA	72.17%
Barron, 2014 [23]	67.4	100%	NA	NA	60.37%	NA
Fraser, 2014 [24]	62	100%	NA	NA	NA	NA
Holmes, 2014 [25]	62	100%	NA	NA	NA	NA
Murray, 2014 [26]	NA	100%	NA	NA	63.21%	NA
Barron, 2015 [27]	65.5	100%	NA	NA	58.62%	NA
Cronin-Fenton, 2016 [28]	Range (29–80)	100%	NA	NA	NA	71.84%
McMenamin, 2017 [29]	NA	NA	NA	NA	NA	NA
Shiao, 2017 [30]	52.2	100%	28.96	NA	NA	NA
Frisk, 2018 [31]	63.1	100%	NA	NA	NA	NA
Strasser-Weippl, 2018 [32]	63.8	100%	94.2	NA	NA	100%
Williams, 2018 [33]	64.9	100%	62	36.13%	NA	NA
Zhou, 2019 [34]	58.9	NA	55.09	NA	NA	NA
Li, 2020 [35]	76.5	NA	NA	NA	NA	NA
McCarthy, 2020 [36]	NA	100%	NA	30.91%	NA	NA
Johns, 2023 [37]	50.4	NA	32.9	NA	NA	NA
Chen, 2024 [38]	52.4	99.50%	84.7	44.20%	NA	81.60%

**Table 3:** Cancer-specific characteristics and treatment details.

First Author, Year	Stage	Grade	HR+ (%)	Her-2 (%)	Positive Lymph Node Status	Received Radiotherapy	Received Chemotherapy	Received Hormone Therapy	Received Surgery
Blair, 2007 [19]	NA	NA	ER (87.18%), PR (72.65%)	NA	28.37%	NA	NA	NA	NA
Holmes, 2010 [20]	Stage I–III, Stage II: 35.98%, Stage III: 6.14%	NA	ER (78.37%)	NA	NA	NA	NA	NA	NA
Wernli, 2011 [21]	NA	NA	NA	NA	NA	47.25%	35.09%	59.35%	98.17%
Li, 2012 [22]	Stage I–IV, Stage I: 57.88%, Stage II: 36.62%, Stage III: 2.9%, Stage IV: 4.17%	NA	ER (70.85%), PR (62.71%)	9%	NA	NA	NA	NA	NA
Barron, 2014 [23]	Stage I–III, Stage I: 30.36%, Stage II: 54.83%, Stage III: 14.81%, Stage IV: 0%	Grade 1–3, Grade 1: 11.59%, Grade 2: 51.45%, Grade 3: 37.00%	ER (79.01%), PR (64.43%)	20%	49.07%	NA	NA	NA	NA
Fraser, 2014 [24]	Stage I–IV, Stage I: 36.58%, Stage II: 42.88%, Stage III: 9.14%, Stage IV: 11.4%	Grade 1–3, Grade 1: 13.21%, Grade 2: 45.65%, Grade 3: 41.14%	ER (78.69%)	NA	19.06%	47.72%	22.91%	70.52%	71.99%
Holmes, 2014 [25]	Stage I–IV, Stage I: 52.3%, Stage II: 40.18%, Stage III: 5.71%, Stage IV: 1.81%	NA	NA	NA	NA	NA	NA	NA	NA
Murray, 2014 [26]	Stage I–IV, Stage I: 64.26%, Stage II: 29.42%, Stage III: 4.68%, Stage IV: 1.64%	Grade 1–3, Grade 1: 16.8%, Grade 2: 35.67%, Grade 3: 47.53%	NA	NA	NA	46.54%	26.22%	NA	83.13%
Barron, 2015 [27]	Stage I–III, Stage I: 32.53%, Stage II: 52.25%, Stage III: 15.22%, Stage IV: 0%	Grade 1–3, Grade 1: 8.93%, Grade 2: 43.99%, Grade 3: 29.22%	ER (83.15%), PR (65.92%)	16.80%	NA	NA	42.49%	73.99%	NA
Cronin-Fenton, 2016 [28]	Stage I–III, Stage I: 37.91%, Stage II: 44.62%, Stage III: 17.47%, Stage IV: 0%	Grade 1–3, Grade 1: 32.19%, Grade 2: 43.91%, Grade 3: 23.90%	ER (79.4%)	NA	NA	43.35%	66.54%	52.85%	100%
McMenamin, 2017 [29]	Stage I–IV, Stage I: 42.44%, Stage II: 40.8%, Stage III: 12.59%, Stage IV: 4.17%	Grade 1–3, Grade 1: 13.19%, Grade 2: 48.92%, Grade 3: 37.89%	ER (83.55%)	NA	NA	36.79%	35.85%	NA	81.90%
Shiao, 2017 [30]	Stage II–III, Stage II: 75.68%, Stage III: 24.32%, Stage IV: 0%	NA	0%	NA	46.12%	80.18%	91.44%	NA	99.10%
Frisk, 2018 [31]	Stage I–IV, Stage I: 56.93%, Stage II: 35.76%, Stage III: 4.49%, Stage IV: 2.82	NA	ER (85.76%)	13.19%	NA	70.22%	39.23%	75.46%	NA
Strasser-Weippl, 2018 [32]	NA	NA	HR (100%)	NA	27.80%	69.50%	31.10%	NA	NA
Williams, 2018 [33]	NA	Grade 1–3, Grade 1: 23.07%, Grade 2: 45.06%, Grade 3: 31.87%	HR (79.72%)	34.79%	41.02%	NA	NA	NA	NA
Zhou, 2019 [34]	NA	Grade 1–3, Grade 1: 21.84%, Grade 2: 31.54%, Grade 3: 41.81%	HR (75.63%)	16.71%	NA	67.48%	41.97%	53.30%	92.26%
Li, 2020 [35]	NA	NA	NA	NA	NA	NA	NA	NA	NA
McCarthy, 2020 [36]	Stage I–III, Stage I: 24.3%, Stage II: 52.19%, Stage III: 23.51%, Stage IV: 0%	Grade 1–3, Grade 1: 10.74%, Grade 2: 55.79%, Grade 3: 33.47%	HR (100%)	NA	NA	NA	80.15%	NA	NA
Johns, 2023 [37]	Stage II–III, Stage II: 38.6%, Stage III: 61.4%, Stage IV: 0%	NA	HR (63.4%)	29.90%	72.80%	NA	100%	62.48%	100%
Chen, 2024 [38]	Stage II–III, Stage II: 68.9%, Stage III: 31.1%, Stage IV: 0%	NA	HR (89%)	0%	89%	NA	NA	NA	83.20%

Abbreviations: ER, Estrogen Receptor; PR, Progesterone Receptor; HR, Hormone Receptor; NA, Not Available.

**Table 4:** Meta-regression analysis.

Variables	*n*	Coefficient	*p*-Value
Disease-Free Survival (DFS)			
Study Publication Year	8	0.027 (−0.042 to 0.096)	0.4379
Mean Age of Study Population	6	0.031 (−0.013 to 0.075)	0.1625
Proportion of Hormone Receptor-Positive	8	0.005 (−0.006 to 0.015)	0.3818
Proportion of HER2-Positive	3	−0.012 (−0.057 to 0.033)	0.5958
Proportion of Postmenopausal Patients	4	0.011 (−0.019 to 0.041)	0.4737
Proportion of Stage I Breast Cancer	6	−0.001 (−0.017 to 0.014)	0.8761
Overall Survival (OS)			
Study Publication Year	18	0.027 (−0.022 to 0.075)	0.2798
Mean Age of Study Population	14	0.010 (−0.018 to 0.038)	0.4904
Proportion of Hormone Receptor-Positive	15	0.004 (−0.007 to 0.015)	0.4308
Proportion of HER2-Positive	7	−0.011 (−0.046 to 0.024)	0.5364
Proportion of Postmenopausal Patients	5	0.010 (−0.025 to 0.046)	0.5652
Proportion of Stage I Breast Cancer	12	0.002 (−0.010 to 0.013)	0.7540
Breast cancer-specific mortality			
Study Publication Year	12	0.068 (−0.011 to 0.147)	0.0906
Mean Age of Study Population	9	0.023 (−0.011 to 0.057)	0.1822
Proportion of Hormone Receptor-Positive	9	−0.009 (−0.040 to 0.021)	0.5525
Proportion of HER2-Positive	5	0.006 (−0.050 to 0.062)	0.8310
Proportion of Postmenopausal Patients	3	−0.003 (−0.043 to 0.037)	0.8786
Proportion of Stage I Breast Cancer	9	0.000 (−0.022 to 0.022)	0.9852

## Data Availability

The data presented in this study are available on request from the corresponding author.

## Supplementary Materials

The following supporting information can be downloaded at: https://www.mdpi.com/article/10.3390/diagnostics15010044/s1, Table S1. PRISMA Checklist; Table S2. Search strategy; Table S3. Reasons for excluded studies; Figure S1. Assessment of risk of bias; Figure S2. Funnel plots and Egger’s test.
