# Supplementary material for: Post-Diagnostic Aspirin Use in Breast Cancer Treatment: A Systematic Review and Meta-Analysis of Survival Outcomes with Trial Sequential Analysis Validation
Source: Diagnostics (Basel). 2024 Dec 27;15(1):44. doi: 10.3390/diagnostics15010044 (PMC11719465; doi:10.3390/diagnostics15010044)

Supplementary material

Post-diagnostic Aspirin Use in Breast Cancer Treatment: A Meta-analysis of Survival Outcomes with Trial Sequential Analysis Validation

Contents

Table S1. PRISMA Checklist

Table S2. Search strategy

Table S3. Reasons for excluded studies

Figure S1. Assessment of risk of bias

Figure S2. Funnel plots and Egger’s test

Table S1. PRISMA Checklist

Section/topic	Item No	Checklist item	Reported on page No
**Title**			
Title	1	Identify the report as a systematic review, meta-analysis, or both	1
**Abstract**			
Structured summary	2	Provide a structured summary including, as applicable, background, objectives, data sources, study eligibility criteria, participants, interventions, study appraisal and synthesis methods, results, limitations, conclusions and implications of key findings, systematic review registration number	1
**Introduction**			
Rationale	3	Describe the rationale for the review in the context of what is already known	3
Objectives	4	Provide an explicit statement of questions being addressed with reference to participants, interventions, comparisons, outcomes, and study design (PICOS)	4
**Methods**			
Protocol and registration	5	Indicate if a review protocol exists, if and where it can be accessed (such as web address), and, if available, provide registration information including registration number	5
Eligibility criteria	6	Specify study characteristics (such as PICOS, length of follow-up) and report characteristics (such as years considered, language, publication status) used as criteria for eligibility, giving rationale	5
Information sources	7	Describe all information sources (such as databases with dates of coverage, contact with study authors to identify additional studies) in the search and date last searched	6
Search	8	Present full electronic search strategy for at least one database, including any limits used, such that it could be repeated	6
Study selection	9	State the process for selecting studies (that is, screening, eligibility, included in systematic review, and, if applicable, included in the meta-analysis)	6
Data collection process	10	Describe method of data extraction from reports (such as piloted forms, independently, in duplicate) and any processes for obtaining and confirming data from investigators	6
Data items	11	List and define all variables for which data were sought (such as PICOS, funding sources) and any assumptions and simplifications made	6
Risk of bias in individual studies	12	Describe methods used for assessing risk of bias of individual studies (including specification of whether this was done at the study or outcome level), and how this information is to be used in any data synthesis	7
Summary measures	13	State the principal summary measures (such as risk ratio, difference in means).	7
Synthesis of results	14	Describe the methods of handling data and combining results of studies, if done, including measures of consistency (such as I^2 statistic) for each meta-analysis	9
Risk of bias across studies	15	Specify any assessment of risk of bias that may affect the cumulative evidence (such as publication bias, selective reporting within studies)	9
Additional analyses	16	Describe methods of additional analyses (such as sensitivity or subgroup analyses, meta-regression), if done, indicating which were pre-specified	10
**Results**			
Study selection	17	Give numbers of studies screened, assessed for eligibility, and included in the review, with reasons for exclusions at each stage, ideally with a flow diagram	10
Study characteristics	18	For each study, present characteristics for which data were extracted (such as study size, PICOS, follow-up period) and provide the citations	10
Risk of bias within studies	19	Present data on risk of bias of each study and, if available, any outcome-level assessment (see item 12).	Supplement information

Section/topic	Item No	Checklist item	Reported on page No
Results of individual studies	20	For all outcomes considered (benefits or harms), present for each study (a) simple summary data for each intervention group and (b) effect estimates and confidence intervals, ideally with a forest plot	10
Synthesis of results	21	Present results of each meta-analysis done, including confidence intervals and measures of consistency	11
Risk of bias across studies	22	Present results of any assessment of risk of bias across studies (see item 15)	Supplement information
Additional analysis	23	Give results of additional analyses, if done (such as sensitivity or subgroup analyses, meta-regression) (see item 16)	Supplement information
**Discussion**			
Summary of evidence	24	Summarise the main findings including the strength of evidence for each main outcome; consider their relevance to key groups (such as health care providers, users, and policy makers)	-
Limitations	25	Discuss limitations at study and outcome level (such as risk of bias), and at review level (such as incomplete retrieval of identified research, reporting bias)	22
Conclusions	26	Provide a general interpretation of the results in the context of other evidence, and implications for future research	22
**Funding**			
Funding	27	Describe sources of funding for the systematic review and other support (such as supply of data) and role of funders for the systematic review	-

Table S2. Search strategy

PubMed:
(("aspirin"[MeSH Terms] OR "aspirin"[Title/Abstract])
OR ("cyclooxygenase inhibitor"[MeSH Terms] OR "COX inhibitor"[Title/Abstract] OR "cyclooxygenase inhibitors"[Title/Abstract]))
AND ("breast cancer"[MeSH Terms] OR "breast carcinoma"[Title/Abstract] OR "breast neoplasms"[MeSH Terms])

EMBASE:
(('aspirin'/exp OR 'aspirin':ti,ab)
OR ('cyclooxygenase inhibitor'/exp OR 'cox inhibitor':ti,ab OR 'cyclooxygenase inhibitors':ti,ab))
AND ('breast cancer'/exp OR 'breast carcinoma':ti,ab OR 'breast neoplasm':ti,ab)

Cochrane:
(([aspirin]:ti,ab,kw OR [acetylsalicylic acid]:ti,ab,kw)
OR ([cyclooxygenase inhibitor]:ti,ab,kw OR [COX inhibitor]:ti,ab,kw OR [cyclooxygenase inhibitors]:ti,ab,kw))
AND ([breast cancer]:ti,ab,kw OR [breast carcinoma]:ti,ab,kw OR [breast neoplasm]:ti,ab,kw)

PubMed Search (2024/10/29): 363 results

Embase Search (2024/10/29): 241 results

Cochrane Library Search (2024/10/29): 143 results

Table S3. Detailed Reasons for Excluded Studies

A supplementary table listing the detailed reasons for excluding studies after full-text assessment. This table provides transparency in the selection process and ensures reproducibility of the systematic review.

Reference	Reason for exclusion
Ratnasinghe LD, Graubard BI, Kahle L, Tangrea JA, Taylor PR, Hawk E. Aspirin use and mortality from cancer in a prospective cohort study. Anticancer Res. 2004;24(5B):3177-3184.	The study does not focus on breast cancer.
Kwan ML, Habel LA, Slattery ML, Caan B. NSAIDs and breast cancer recurrence in a prospective cohort study. Cancer Causes Control. 2007;18(6):613-620. doi:10.1007/s10552-007-9003-y	The article lacks relevant survival data (e.g., Relative Risk, RR).
Zhang SM, Cook NR, Manson JE, Lee IM, Buring JE. Low-dose aspirin and breast cancer risk: results by tumour characteristics from a randomised trial. Br J Cancer. 2008;98(5):989-991. doi:10.1038/sj.bjc.6604240	The article only addresses breast cancer incidence and is not applicable for survival analysis.
Rothwell PM, Fowkes FG, Belch JF, Ogawa H, Warlow CP, Meade TW. Effect of daily aspirin on long-term risk of death due to cancer: analysis of individual patient data from randomised trials. Lancet. 2011;377(9759):31-41. doi:10.1016/S0140-6736(10)62110-1	The study does not focus on breast cancer.
Rothwell PM, Wilson M, Price JF, Belch JF, Meade TW, Mehta Z. Effect of daily aspirin on risk of cancer metastasis: a study of incident cancers during randomised controlled trials. Lancet. 2012;379(9826):1591-1601. doi:10.1016/S0140-6736(12)60209-8	The article provides data on pre-diagnostic aspirin use, which does not align with the purpose of post-diagnostic analysis.
Sendur MA, Aksoy S, Ozdemir NY, Zengin N, Altundag K. Impact of acetylsalicylic Acid on the clinicopathological characteristics and	The article provides data on pre-diagnostic aspirin use, which does

Reference	Reason for exclusion
prognosis of patients with invasive breast cancer. Breast Care (Basel). 2014;9(4):261-266. doi:10.1159/000365952	not align with the purpose of post-diagnostic analysis.
Bradley MC, Black A, Freedman AN, Barron TI. Prediagnostic aspirin use and mortality in women with stage I to III breast cancer: A cohort study in the Prostate, Lung, Colorectal, and Ovarian Cancer Screening Trial. Cancer. 2016;122(13):2067-2075. doi:10.1002/cncr.30004	The article provides data on pre-diagnostic aspirin use, which does not align with the purpose of post-diagnostic analysis.
Wang T, Parada H, McClain KM, et al. Pre-diagnostic aspirin use and mortality after breast cancer. Cancer Causes Control. 2018;29(4-5):417-425. doi:10.1007/s10552-018-1020-5	The article provides data on pre-diagnostic aspirin use, which does not align with the purpose of post-diagnostic analysis.
Loomans-Kropp HA, Pinsky P, Umar A. Evaluation of Aspirin Use With Cancer Incidence and Survival Among Older Adults in the Prostate, Lung, Colorectal, and Ovarian Cancer Screening Trial. JAMA Netw Open. 2021;4(1):e2032072. Published 2021 Jan 4. doi:10.1001/jamanetworkopen.2020.32072	The article provides data on pre-diagnostic aspirin use, which does not align with the purpose of post-diagnostic analysis.
Oghazian MB, Shirzad N, Ahadi M, Eivazi Adli S, Mollazadeh S, Radfar M. Aspirin versus placebo on estrogen levels in postmenopausal women: a double-blind randomized controlled clinical trial. BMC Pharmacol Toxicol. 2022;23(1):31. Published 2022 May 17. doi:10.1186/s40360-022-00571-9	The study does not focus on breast cancer.

Figure S1. Assessment of risk of bias (RoB 2 for RCT and ROBINS-I for non-randomized studies of interventions)

RoB 2 for RCT

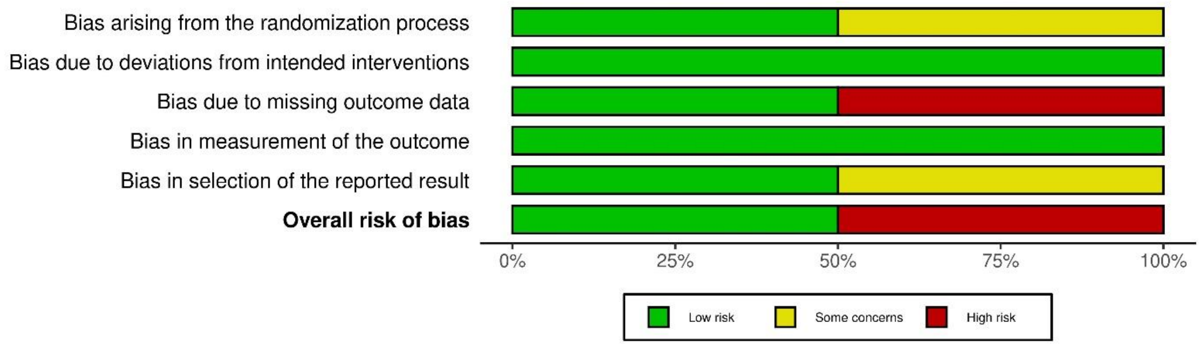

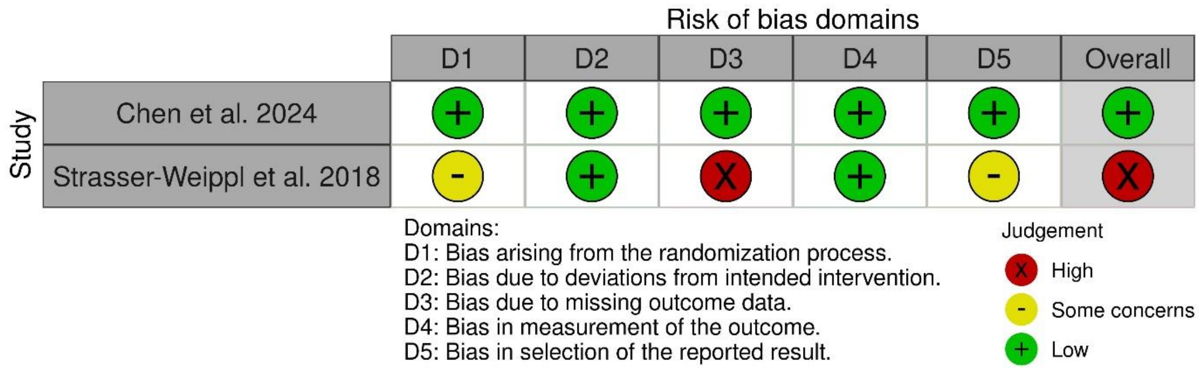

ROBINS-I for non-randomized studies of interventions

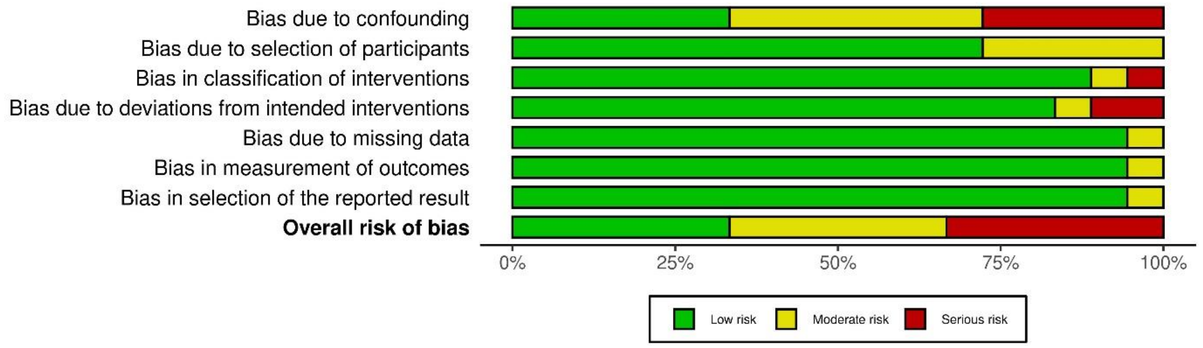

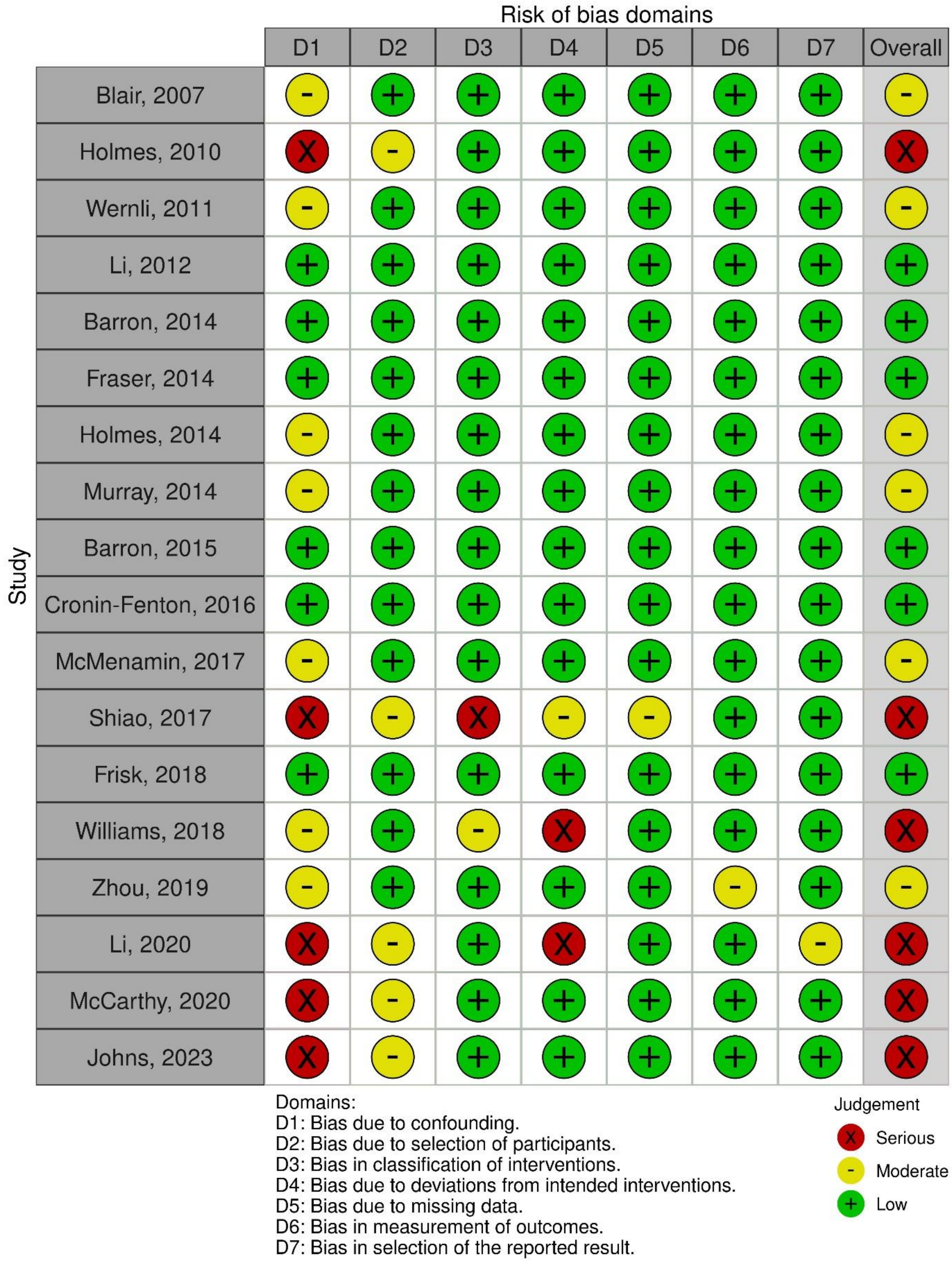
Risk of bias domains
D1
D2
D3
D4
D5
D6
D7
Overall
Study
Blair, 2007
Holmes, 2010
Wernli, 2011
Li, 2012
Barron, 2014
Fraser, 2014
Holmes, 2014
Murray, 2014
Barron, 2015
Cronin-Fenton, 2016
McMenamin, 2017
Shiao, 2017
Frisk, 2018
Williams, 2018
Zhou, 2019
Li, 2020
McCarthy, 2020
Johns, 2023
Domains:
D1: Bias due to confounding.
D2: Bias due to selection of participants.
D3: Bias in classification of interventions.
D4: Bias due to deviations from intended interventions.
D5: Bias due to missing data.
D6: Bias in measurement of outcomes.
D7: Bias in selection of the reported result.
Judgement
Serious
Moderate
Low

Figure S2. Funnel plots and Egger's test

DFS

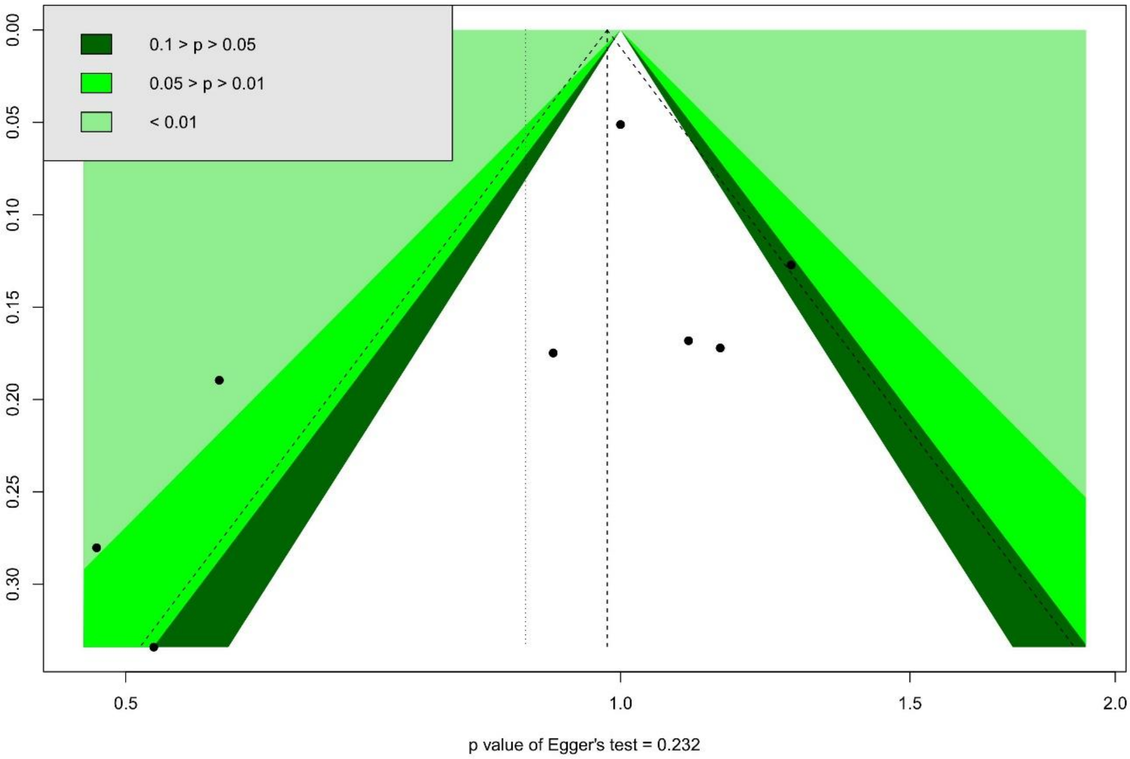

OS

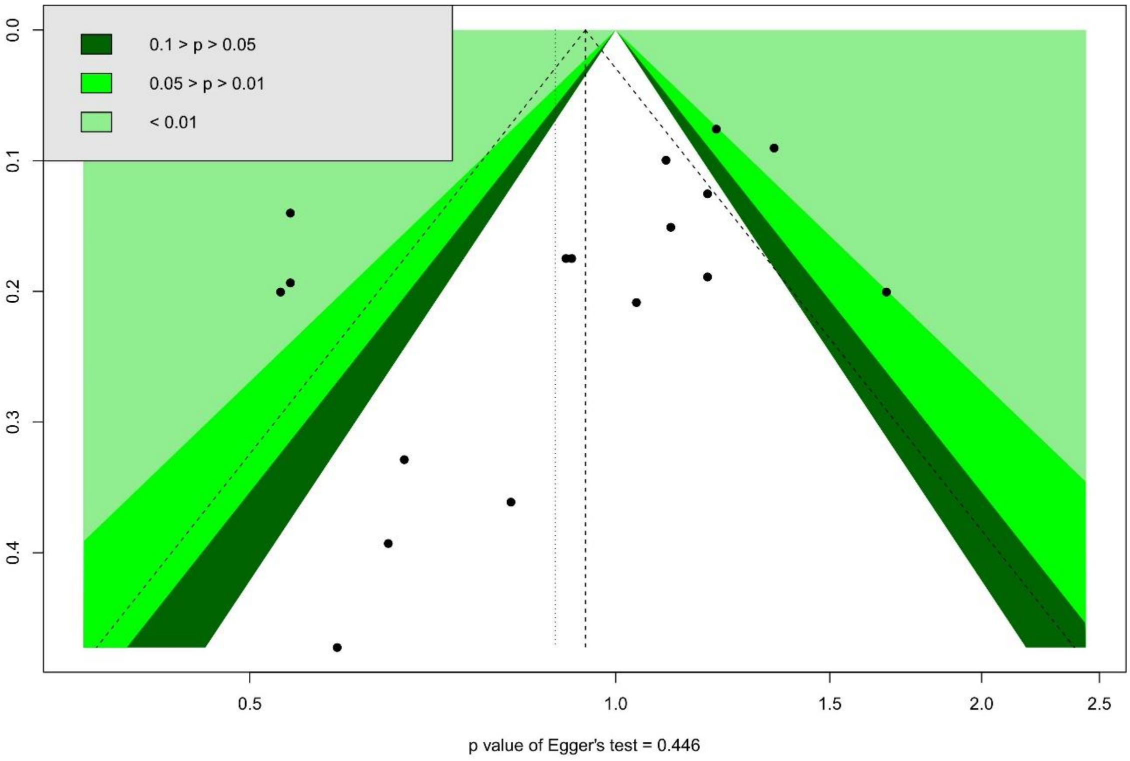

Breast cancer specific OS

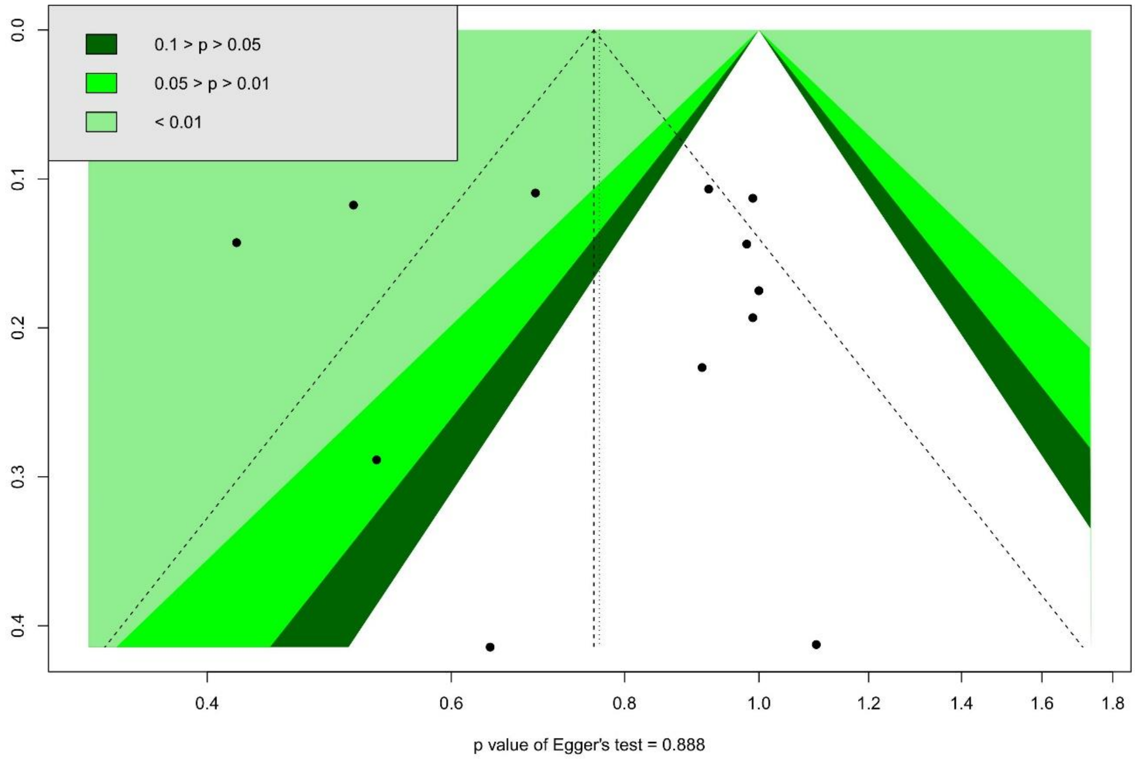

Supplement: Supplementary file 1 [file diagnostics-15-00044-s001.zip › diagnostics-3375286-supplementary.pdf]
